# Estimation and change of edentulism among the Korean population: Korea National Health and Nutrition Examination Survey 2007-2018

**DOI:** 10.4178/epih.e2021020

**Published:** 2021-04-02

**Authors:** Na-Hyeon Yu, Ah Ra Shin, Song Vogue Ahn, Keun-Bae Song, Youn-Hee Choi

**Affiliations:** 1 Department of Preventive Dentistry, School of Dentistry, Kyungpook National University, Daegu, Korea; 2 Department of Dental Hygiene, Gumi University, Gumi, Korea; 3 Department of Health Convergence, Ewha Womans University, Seoul, Korea

**Keywords:** Oral health, Edentulism, Prevalence, Tooth loss, Korea National Health and Nutrition Examination Survey

## Abstract

**OBJECTIVES:**

To estimate the prevalence of edentulism according to the socio-demographic variables of Korean adults between 2007 and 2018 and to analyze the trends.

**METHODS:**

This study was conducted using raw data from the Korea National Health and Nutrition Examination Survey (KNHANES) from 2007 to 2018. Edentulism was defined as the absence of upper and lower teeth or the retained root of the missing teeth. To obtain the prevalence of edentulism, complex sample frequency analysis and regression analysis were performed according to the socio-demographic variables to represent the national population. The Korean population structure in 2005 was exploited as a standard population to calculate age-standardized edentulism.

**RESULTS:**

The prevalence of edentulism in 2016-2018 was 9.7% in the Korean elderly, and the change of age-standardized edentulism steadily declined over time from 2007, 12.8%. In particular, it decreased by approximately 20% in the period between 2016 and 2018 compared to that in 2007 for those in their 80s. The trend of the prevalence according to gender decreased significantly in women. According to the level of education, the greatest decrease was seen in the group with the lowest education, although the prevalence itself was higher than that seen in those who were more educated.

**CONCLUSIONS:**

The prevalence of edentulism among the older Korean population has decreased over time. However, the concern is on those with lower education and men as these groups are still at higher risk for edentulism.

## INTRODUCTION

Globally total tooth loss is estimated to affect of oral of burden 267 million individuals in 2017 [1], and has significant negative effects on the quality of life and oral and general health [2]. It leads to the deterioration of the basic functions of teeth such as mastication and speech and a loss of aesthetic sense [3], which further causes weight loss, nutritional loss, and systemic diseases. In severe cases, it also leads to death [4-7]. Therefore, maintaining oral health is essential to maintain systemic health. Tooth loss generally starts around the age of 40 and becomes rapidly prevalent after the age of 65 [8]. It is caused by various factors such as dental caries, periodontal disease, tumors in the jawbone, and trauma [9,10]. Functional loss caused by tooth loss can be restored through prosthetic treatment.

Edentulism refers to a condition in which all natural teeth have been lost without any prosthetic treatment. Although prevalence differs by country and region, it is generally prevalent in adults over the age of 50. It is also observed in younger age groups. Thus, it is one of the many health problems that requires care and management [11-13]. The prevalence of edentulism for the last 30 years has decreased in developed countries (e.g., United States); however, low-income families and the elderly still suffer from masticatory problems due to edentulism [13,14].

With the recent aging of the general population, the interest in the relationship between the elderly and oral health has increased worldwide. Research on the trend of edentulism prevalence is actively being conducted in many different countries, with comparisons between the countries as well. For example, Cardoso et al. [15] assessed the prevalence and trend of edentulism using data from Brazil’s national oral health survey, and Eklund & Burt [16] used the Unite States national survey data to investigate the prevalence of edentulism. Additionally, Elani et al. [17] compared the education-based social inequality for the prevalence of edentulism and tooth loss between five countries (Australia, Canada, Chile, New Zealand, and United States). In 2016, Jun & Ryu [3] reported the oral health behavior of the elderly according to tooth loss using raw data from the 6th National Health and Nutrition Survey in Korea. However, there is a lack of epidemiological studies on the prevalence of edentulism and rate of tooth loss in Korea even though the elderly over the age of 65 accounts for the 14.9% of the total population as of 2019.

Eklund & Burt [16] analyzed the trend of edentulism prevalence according to demographic variables, which are important predictors of edentulism prevalence, and reported that tooth loss was more frequent in the low-education and low-income groups despite the decrease in the overall prevalence of edentulism. In Korea, Ryu & Jeon [18] showed that low-income groups who are eligible for medical benefits have a lower rate of receiving implants that have been covered by insurance since 2014, compared to local/workplace subscribers. However, studies on the prevalence of edentulism according to demographic variables before and after insurance coverage are not reported.

Studies on the prevalence of edentulism can help assess and in the diagnosis of the oral health of the population and help establish policies for improvement. Therefore, the purpose of this study was to estimate the prevalence of edentulism in Korea according to demographic variables for each year using raw data from the 2007-2018 (4th-7th) KNHANES and to determine differences in the trend of edentulism prevalence change.

## MATERIALS AND METHODS

### Research subjects

In this study, raw data from the 4th (2007-2009), 5th (2010-2012), 6th (2013-2015), and 7th (2016-2018) KNHANES, a representative health and nutritional survey in Korea, were analyzed.

Data from a total of 63,791 adult subjects aged 19 and older, including 2,925 in 2007, 6,681 in 2008, 7,403 in 2009, 6,206 in 2010, 6,039 in 2011, 5,757 in 2012, 5,349 in 2013, 5,118 in 2014, 5,024 in 2015, and 13,199 in 2016-2018, were used. The Korea Centers for Disease Control and Prevention provided raw data for each year from the 4th to the 6th survey. However, data from the 7th survey was combined for three years (2016-2018) because the complete enumeration survey could not be conducted due to the lack of public health dentists who are required for oral examinations [19].

### Methods

#### Demographic variables

Demographic variables included age, gender, income level, and education level. Patients were classified by age into the following groups: 19 and over, 65 and over, 30s, 40s, 50s, 60s, 70s, and 80s and over. This was based on age standardization using the estimated population ratio reported in 2005. Gender was categorized into men and women. Income level was divided into “poorest”, “poor”. “rich”, and “richest”, while education level was categorized into “none or elementary school”, “middle school”, “high school”, and “college or university”.

#### Oral examination

The oral examination in the KNHANES was conducted in two parts: questionnaire survey and medical examination survey, which were conducted at mobile examination centers by public health dentists who received prior education and training. Examination items included the condition of the teeth and prosthesis and the need for treatment, based on the World Health Organization oral examination guidelines. The surface condition code and treatment need code were divided from 0 to 9 [19].

#### Definition of edentulism

The operational definition of edentulism is the absence of any teeth in the oral cavity [20]. In this study, edentulism was defined by the absence of residual teeth in the upper and lower jaws and retained root of the missing teeth to analyze and compare the prevalence of edentulism.

### Statistical analysis

KNHANES data have a complex sample design. Thus, statistical analysis was performed by applying the weights suggested by the Korea Centers for Disease Control and Prevention for each year. Based on the estimated population in 2005, age was standardized by dividing the age groups into 19-29, 30-39, 40-49, 50-59, 60-69, and 70 or older. Additionally, those values without age standardization were also presented to compare with the actual prevalence rates for the relevant year.

Complex sample frequency analysis was conducted to compare the general characteristics of patients and distribution of edentulism for each year. Moreover, regression analysis was performed for the prevalence of edentulism according to demographic variables for each year to evaluate the trend of edentulism prevalence by year.

A p-value less than 0.05 was considered statistically significant, and statistical analysis was performed using SAS version 9.4 (SAS Institute, Cary, NC, USA).

### Ethics statement

According to Article 2, No.1 of the Bioethics Act and the Enforcement Regulations of the same Act, Article 2, No. 2.1, KNHANES is research conducted directly by the nation for public welfare. Therefore, it can be conducted without the approval of the institutional review board.

## RESULTS

### Trend of edentulism prevalence according to age

**Figure 1.**
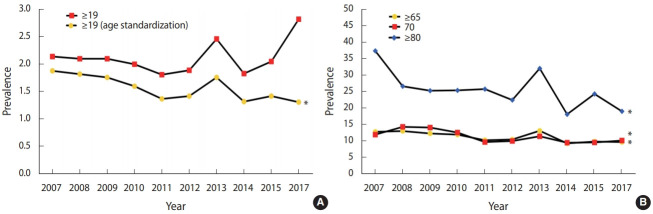
Trend analysis of edentulism in Korea National Health and Nutrition Examination Survey by age (A: ≥19 yr, B: ≥65 yr). ^*^p<0.05 for trend test.

The trend of edentulism prevalence was analyzed according to age. In all subjects over the age of 19 who were age-standardized, the prevalence of edentulism decreased in general after 2007 and showed a slight increase in 2012 and 2013, followed by a decline (p< 0.05) (Figure 1A). In the elderly over the age of 65 as well, edentulism also showed the trend of slight increase and decrease from 2007, with an overall decrease in the prevalence by 2017 compared to that in 2007 (p < 0.05) (Figure 1B). In the elderly over the age of 80, edentulism prevalence was 40% in 2007 and decreased significantly by 2017 (p< 0.05) (Figure 1B). Although the trend of edentulism by age varied in magnitude, an overall decreasing trend was observed over time, regardless of age, compared to that in 2007. Additionally, the higher the age, the higher the prevalence of edentulism. In all groups, edentulism slightly increased in 2013, followed by a decreasing trend. In the elderly over the age of 80, the magnitude of the increase and decrease in 2013 was larger compared to that of the other age groups (p < 0.05) (Figure 1).

### Trend of edentulism prevalence according to gender

**Figure 2.**
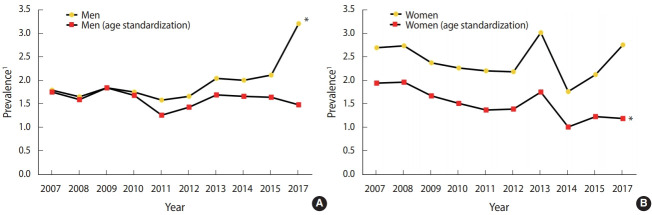
Trend analysis of edentulism in Korea National Health and Nutrition Examination Survey by gender (A: men, B: women). ^1^ Prevalence of 2017 was calculated from 2016-2018 data; crude prevalence means real magnitude of edentulism at the time of the survey. *p<0.05 for trend test.

Figure 2 shows the trend of edentulism according to gender. In men without age standardization, the prevalence of edentulism appeared to significantly increase. In contrast, after age standardization, there was a repeat of increase and decrease, with an overall decreasing trend in the prevalence of edentulism (Figure 2A). Similarly, the prevalence of edentulism in women also showed a repeat of increase and decrease. However, the prevalence was significantly decreased compared to that of men. The trend of edentulism prevalence was similar in women with and without age standardization, and a repeat of slight increase and decrease was observed between 2007 and 2017 (p< 0.05) (Figure 2B).

### Analysis of edentulism prevalence trend

Table 1 shows the result of analyzing the prevalence of edentulism according to age and gender. The prevalence of edentulism in those over the age of 19 after standardization was 1.88% in 2007 and had an overall decreasing trend. In 2017, it was slightly decreased to 1.31% (p< 0.05). In the elderly over the age of 65, the prevalence of edentulism showed a repeat of increase and decrease. In 2016-2018, the prevalence decreased by 25% to 9.72% compared to 12.80% in 2007 (p<0.05). The prevalence of edentulism increased with increasing age. However, in all age groups, the prevalence decreased in 2017 compared to that in 2007. In particular, the prevalence in those over the age of 80 decreased significantly by 50% from 37.30% to 19.01% (p< 0.05). Additionally, the prevalence was slightly increased in 2013 and decreased again except in those aged in their 60s. In the 80s group, the prevalence showed a large increase and decrease in 2013 compared to that in other age groups (p< 0.05).

The prevalence of edentulism showed a trend of repeated increase and decrease between 2007 and 2017 in both men and women. In 2007, the prevalence after age standardization was 1.75% in men, which was lower than the 1.94% seen in in women. However, in 2017, the prevalence of edentulism was 1.48% in men, which was higher than the 1.19% observed in women. Moreover, there was a significant change in the prevalence of edentulism in women (p< 0.05) (Table 1).

The prevalence of edentulism was analyzed according to income level after age standardization. In 2007, the difference between the groups was not significant. However, the prevalence decreased by 50% in 2017 compared to that in 2007 in the group with the highest socioeconomic status. The magnitudes of increase and decrease in the prevalence between 2007 and 2017 were higher in lower groups than higher groups of income level (Table 2).

The prevalence of edentulism was analyzed according to education level after age standardization (p< 0.05). In the “none or elementary school” group, the prevalence decreased by more than 50% from 2.97% in 2007 to 1.34% in 2017; however, the prevalence was still higher compared to that of the other groups (p< 0.05). In 2007, the difference in the prevalence of edentulism between the “none or elementary group” and “college or university” group was 2.37%, which decreased over time to 0.76% by 2017 (Table 2).

## DISCUSSION

Tooth loss is an indicator of oral health; currently, studies are evaluating the trend of edentulism prevalence as well as making comparisons between different countries in this regard [8,17]. However, there is a lack of studies on the prevalence of edentulism in Korea, one of the main Asian countries. Therefore, this study was conducted to understand the trend of edentulism among adults over the age of 19 using raw data from the 2007-2018 (4th-7th) KNHANES.

In this study, the trend of edentulism according to demographic variables showed a repeated trend of increase and decrease, with an overall decrease in prevalence by 2016-2018 compared to that in 2007. Moreover, edentulism prevalence increased with aging, and the magnitude in decrease of prevalence was also greater. We observed that in the 80s group, the prevalence increased by 9.57% from 2012 to 2013 and decreased by 13.89% from 2013 to 2014. This coincides with the time when implants started to be covered by health insurance for those citizens over the age of 75 in July 2014. Ryu & Jeon [18] reported that the rate of use of implant coverage increased from 1.2% in 2014 to 3.2% in 2015 and 4.9% in 2016. When there are not enough teeth to serve as abutment teeth for removable partial dentures, the remaining teeth are removed, and implants are installed in order to use removable partial dentures. In such cases, it is thought that insurance coverage for implants would have affected the decrease in the prevalence of edentulism. In 2016-2018, the magnitude of the decrease in edentulism prevalence in women was significant compared to that in 2007. However, in 2014 when implants started to be covered by insurance benefits, the prevalence of edentulism decreased in men while it increased in women. This may also be related to the higher rate of usage of implant coverage by men than by women [18]. When the prevalence was analyzed according to income level, the magnitude in the decrease of edentulism was greater in groups with a higher socioeconomic status than in those with lower socioeconomic status. Additionally, the groups with lower socioeconomic status had a higher prevalence of edentulism than those belonging to the higher status groups. This finding is consistent with the finding of Eklund & Burt [16] who reported that the elderly are highly influenced by socioeconomic factors of edentulism and that the chance of tooth loss in low-income families is high despite the decrease in the overall prevalence of edentulism. Moreover, our results are in line with those of Ryu & Jeon [18] who reported that the rate of usage of implant coverage by National Health Insurance was lower than that of local/work-based subscribers. Although the implant may be covered by insurance, there are many items of dental treatment that are not part of the medical benefits that would have affected the high prevalence of edentulism in low-income groups. Our finding on the prevalence of edentulism according to education level is similar to the results of a study by Elani et al. [17] on the comparison of the prevalence in five different countries. We observed that the prevalence of edentulism was lower in the high education level groups than in the low education level groups.

As an additional analysis, the teeth with severe mobility or root rest were treated as tooth loss and then edentulism was re-calculated. We obtained almost similar results. However, the prevalence of edentulism in 2016-2018 was 0.43% higher in elderly people over the age of 80 compared to the result of the present study. Older persons may be prone to have the dysfunctional teeth left untreated in their mouth (data not shown). It is thought to be related to the rate of limited oral function, which is caused by problems in the oral cavity, such as in the teeth and gums, and leads to mastication and speech discomfort. In fact, 2009-2019 KNHANES demonstrated that the rate of limited oral function increases with age [21].

We observed that a higher age and lower income and education level led to a higher chance of tooth loss and higher prevalence of edentulism. This finding is consistent with the results of previous studies conducted in other countries [8,22,23]. Although there are differences in the prevalence according to demographic variables, the overall decrease in prevalence over time is thought to have affected the expansion of the age for implants and the reduction in the self-pay rate. In addition, a previous study on the subjective oral health status in the elderly in Korea using data of KNHANES showed that the number of elderly persons who report bad subjective oral health is decreasing. It is possible that increased interest in oral health had positive effects on the prevalence of edentulism [24]. Among Organization for Economic Cooperation and Development countries, the health level of the Korean general population is comparable to that of other countries. However, in those having relatively fewer medical benefits with difficulty in access of medical care have less medical use as called Inverse Care Law, dental care system still needs to be improved in Korea [25]. Considering that there is still a gap in the prevalence of edentulism according to income and education levels, universal policies such as increased access to oral health and increased benefits for the low-income and low-educated groups would need to be continuously pursued.

There are several limitations in this study. Although data from KNHANES were used, the oral survey was a cross-sectional study with oral examinations and questionnaires. Therefore, the cause of the increase in the prevalence of edentulism in all groups, except the 60s, in 2013 could not be identified. Therefore, further studies would be required. In addition, other factors related to tooth loss, such as alcohol consumption and smoking, were not considered in this study. Lastly, the vulnerable population living in nursing homes, long-term care hospitals, and rehabilitation facilities were not included in KNHANES.

Nonetheless, this is the first study to analyze the trend of edentulism prevalence in Korea using large-scale samples of KNHANES, which represents the Korean population. Moreover, this study can be used as a reference material for the Korean population in future studies comparing the prevalence of edentulism among different countries.

In conclusion, the prevalence of edentulism in those over the age of 65 was generally decreased from 12.80% to 9.72% over time. There were also significant changes in the trend of edentulism in the different age groups according to demographic factors such as age, gender, income level, and education level. The prevalence of edentulism is higher in the elderly and in those with low income and education level. The introduction of expanded insurance benefit for implants in 2014 is expected to contribute to the decrease in edentulism. However, there are still other groups that suffer from edentulism and, thus, follow-up studies would be required. We expect that the study results could be used as basic data on oral health related to tooth loss and edentulism.

## Figures and Tables

**Table 1.** Changes in edentulism prevalence^1^ according to age and gender

Variables		2007	2008	2009	2010	2011	2012	2013	2014	2015	2017^2^	Trend test p-value
Age (yr)												
	≥19	2,925 (2.14) [0.35]	6,681 (2.10) [0.19]	7,403 (2.10) [0.19]	6,206 (2.00) [0.21]	6,039 (1.81) [0.21]	5,757 (1.89) [0.21]	5,439 (2.46) [0.24]	5,118 (1.83) [0.20]	5,024 (2.05) [0.20]	13,199 (2.82) [0.23]	0.241
	≥19 (standardization)	2,925 (1.88) [0.26]	6,681 (1.82) [0.14]	7,403 (1.76) [0.14]	6,206 (1.60) [0.15]	6,039 (1.37) [0.14]	5,757 (1.42) [0.13]	5,439 (1.76) [0.15]	5,118 (1.32) [0.14]	5,024 (1.42) [0.13]	13,199 (1.31) [0.09]	0.007
	≥65	727 (12.80) [1.92]	1,497 (13.04) [1.11]	1,658 (12.31) [1.05]	1,396 (11.95) [1.27]	1,521 (10.26) [1.05]	1,559 (10.48) [1.13]	1,289 (13.13) [1.15]	1,399 (9.30) [0.93]	1,349 (9.85) [0.99]	3,426 (9.72) [0.72]	0.014
	50s	494 (1.27) [0.58]	1,116 (1.06) [0.33]	1,235 (1.03) [0.36]	1,164 (1.03) [0.26]	1,168 (1.22) [0.37]	1,087 (0.65) [0.31]	1,021 (1.24) [0.42]	967 (0.85) [0.37]	1,029 (1.09) [0.49]	2,493 (0.58) [0.21]	0.094
	60s	479 (4.13) [0.96]	1,063 (4.35) [0.77]	1,196 (4.05) [0.68]	1,011 (3.45) [0.72]	1,033 (2.43) [0.62]	1,028 (4.15) [0.73]	870 (3.63) [0.72]	890 (3.37) [0.62]	936 (2.75) [0.66]	2,256 (3.08) [0.44]	0.061
	70s	371 (11.95) [1.64]	754 (14.31) [1.52]	859 (14.10) [1.60]	734 (12.58) [1.57]	828 (9.71) [1.18]	879 (10.02) [1.45]	669 (11.45) [1.54]	733 (9.51) [1.32]	697 (9.59) [1.41]	1,746 (10.16) [0.93]	0.017
	≥80s	87 (37.30) [7.89]	195 (26.59) [4.04]	205 (25.25) [3.48]	159 (25.35) [4.41]	206 (25.73) [3.92]	198 (22.42) [3.78	201 (31.99) [3.84]	220 (18.10) [2.73]	200 (24.27) [3.22]	621 (19.01) [1.76]	0.042
Gender												
	Men	1,215 (1.79) [0.36]	2,787 (1.65) [0.25]	3,207 (1.84) [0.25]	2,678 (1.75) [0.27]	2,569 (1.58) [0.23]	2,389 (1.66) [0.28]	2,336 (2.04) [0.26]	2,133 (2.00) [0.28]	2,187 (2.11) [0.29]	5,793 (3.20) [0.31]	0.014
	Men (standardization)	1,215 (1.75) [0.34]	2,787 (1.59) [0.22]	3,207 (1.84) [0.22]	2,678 (1.68) [0.23]	2,569 (1.26) [0.19]	2,389 (1.43) [0.22]	2,336 (1.69) [0.21]	2,133 (1.66) [0.22]	2,187 (1.64) [0.21]	5,793 (1.48) [0.14]	0.385
	Women	1,710 (2.69) [0.52]	3,894 (2.73) [0.28]	4,196 (2.37) [0.27]	3,528 (2.26) [0.28]	3,470 (2.20) [0.32]	3,368 (2.18) [0.26]	3,103 (3.01) [0.38]	2,985 (1.76) [0.24]	2,837 (2.12) [0.32]	7,406 (2.75) [0.30]	0.624
	Women (standardization)	1,710 (1.94) [0.32]	3,894 (1.96) [0.18]	4,196 (1.67) [0.18]	3,528 (1.51) [0.18]	3,470 (1.37) [0.17]	3,368 (1.39) [0.16]	3,103 (1.75) [0.20]	2,985 (1.01) [0.13]	2,837 (1.23) [0.19]	7,406 (1.19) [0.12]	0.005

Values are presented as unweighted number (% weighted proportion) [standard error].

1 Crude prevalence means real magnitude of edentulism at the time of the survey.

2 Analyzed from the 2016-2018 data.

**Table 2.** Changes in edentulism prevalence^1^ according to income level and education level

Variables			2007	2008	2009	2010	2011	2012	2013	2014	2015	2017^2^	Trend test p-value
Comparison of income level according to standardization													
	Income level												
		Poorest	616 (6.21) [0.93]	1,368 (7.45) [0.77]	1,581 (6.48) [0.69]	1,252 (6.37) [0.90]	1,212 (7.14) [0.83]	1,104 (5.96) [0.82]	1,120 (8.26) [0.86]	1,034 (6.15) [0.80]	996 (6.70) [0.86]	2,641 (9.19) [0.84]	0.161
		Poor	727 (1.84) [0.47]	1,689 (1.50) [0.28]	1,707 (2.49) [0.45]	1,551 (1.54) [0.35]	1,553 (1.27) [0.32]	1,469 (2.18) [0.39]	1,419 (1.38) [0.32]	1,277 (2.26) [0.47]	1,207 (2.73) [0.54]	3,209 (2.70) [0.34]	0.112
		Rich	704 (0.63) [0.24]	1,692 (0.97) [0.26]	2,007 (0.78) [0.19]	1,680 (0.89) [0.27]	1,635 (0.58) [0.15]	1,483 (0.39) [0.13]	1,368 (1.48) [0.32]	1,429 (0.75) [0.20]	1,351 (0.77) [0.24]	3,602 (1.13) [0.20]	0.417
		Richest	726 (0.90) [0.34]	1,722 (0.94) [0.23]	2,020 (0.50) [0.16]	1,639 (0.68) [0.18]	1,579 (0.69) [0.22]	1,615 (0.79) [0.27]	1,492 (1.11) [0.32]	1,351 (0.47) [0.21]	1,436 (0.42) [0.17]	3,709 (0.57) [0.13]	0.213
	Income level (standardization)												
		Poorest	616 (1.75) [0.25]	1,368 (2.39) [0.28]	1,581 (1.84) [0.20]	1,252 (1.90) [0.26]	1,212 (1.91) [0.31]	1,104 (1.35) [0.19]	1,120 (2.27) [0.33]	1,034 (1.30) [0.17]	996 (1.92) [0.36]	2,641 (1.72) [0.17]	0.399
		Poor	727 (1.95) [0.48]	1,689 (1.36) [0.25]	1,707 (2.07) [0.34]	1,551 (1.56) [0.34]	1,553 (1.18) [0.25]	1,469 (1.65) [0.28]	1,419 (1.01) [0.24]	1,277 (1.57) [0.31]	1,207 (1.82) [0.38]	3,209 (1.24) [0.17]	0.287
		Rich	704 (1.07) [0.42]	1,692 (1.62) [0.40]	2,007 (1.29) [0.31]	1,680 (1.34) [0.40]	1,635 (0.78) [0.23]	1,483 (0.58) [0.18]	1,368 (1.96) [0.39]	1,429 (0.98) [0.25]	1,351 (0.87) [0.25]	3,602 (0.93) [0.16]	0.425
		Richest	726 (1.39) [0.60]	1,722 (1.87) [0.39]	2,020 (0.99) [0.26]	1,639 (1.08) [0.29]	1,579 (1.16) [0.35]	1,615 (1.18) [0.37]	1,492 (1.85) [0.51]	1,351 (0.66) [0.25]	1,436 (0.62) [0.25]	3,709 (0.77) [0.18]	0.089
Comparison of education level according to standardization													
	Education level												
		None or elementary school	921 (8.02) [1.30]	1,981 (8.87) [0.75]	2,052 (7.80) [0.68]	1,568 (6.79) [0.81]	1,545 (7.50) [0.88]	1,424 (6.01) [0.77]	1,226 (8.62) [1.00]	1,120 (5.98) [0.78]	1,095 (7.46) [0.82]	2,629 (8.98) [0.76]	0.875
		Middle school	315 (1.83) [0.69]	749 (1.91) [0.51]	820 (1.84) [0.52]	662 (2.18) [0.54]	655 (1.46) [0.46]	590 (1.96) [0.53]	526 (2.20) [0.69]	496 (2.51) [0.68]	510 (3.77) [1.17]	1,233 (3.58) [0.63]	0.006
		High school	942 (0.53) [0.22]	2,253 (0.13) [0.06]	2,560 (0.42) [0.11]	2,028 (0.46) [0.16]	1,954 (0.28) [0.10]	1,815 (0.69) [0.26]	1,780 (0.88) [0.24]	1,518 (0.74) [0.18]	1,522 (0.59) [0.19]	4,028 (1.20) [0.20]	0.008
		College or university	703 (0.10) [0.08]	1,674 (0.35) [0.17]	1,911 (0.19) [0.12]	1,868 (0.25) [0.09]	1,746 (0.32) [0.14]	1,633 (0.06) [0.03]	1,571 (0.31) [0.13]	1,467 (0.39) [0.20]	1,498 (0.35) [0.15]	4,676 (0.36) [0.10]	0.124
	Education level (standardization)												
		None or elementary school	921 (2.97) [0.48]	1,981 (2.25) [0.20]	2,052 (1.98) [0.20]	1,568 (1.57) [0.19]	1,545 (1.79) [0.26]	1,424 (2.56) [0.31]	1,226 (1.93) [0.28]	1,120 (1.19) [0.16]	1095 (1.55) [0.23]	2,629 (1.34) [0.12]	0.021
		Middle school	315 (1.62) [0.56]	749 (1.13) [0.34]	820 (1.38) [0.37]	662 (1.34) [0.35]	655 (0.82) [0.27]	590 (0.97) [0.25]	526 (1.03) [0.32]	496 (1.19) [0.28]	510 (2.14) [0.99]	1,233 (1.13) [0.22]	0.936
		High school	942 (1.73) [0.61]	2,253 (0.42) [0.22]	2,560 (1.10) [0.32]	2,028 (1.22) [0.42]	1,954 (0.65) [0.26]	1,815 (1.19) [0.42]	1,780 (1.26) [0.33]	1,518 (1.33) [0.32]	1,522 (0.80) [0.24]	4,028 (1.04) [0.18]	0.836
		College or university	703 (0.60) [0.43]	1,674 (0.62) [0.26]	1,911 (0.49) [0.23]	1,868 (0.92) [0.39]	1,746 (0.97) [0.48]	1,633 (0.18) [0.09]	1,571 (0.83) [0.36]	1,467 (0.64) [0.27]	1,498 (1.08) [0.44]	4676 (0,.58) [0.15]	0.650

Values are presented as unweighted number (% weighted proportion)/[standard error].

1 Crude prevalence means real magnitude of edentulism at the time of the survey.

2 Analyzed from the 2016-2018 data.

## SUPPLEMENTARY MATERIALS

Korean version is available at http://www.e-epih.org/.
