# Supplementary material for: Estimation and change of edentulism among the Korean population: Korea National Health and Nutrition Examination Survey 2007-2018
Source: Epidemiol Health. 2021 Apr 2;43:e2021020. doi: 10.4178/epih.e2021020 (PMC8189846; doi:10.4178/epih.e2021020)
Supplement: Supplementary file 1 [file epih-43-e2021020-suppl1.docx]

**Estimation and change of edentulism among the Korean population: Korea National Health and Nutrition Examination Survey 2007-2018**

Na-Hyeon Yu^1,†^, Ah Ra Shin^2,†^, Song Vogue Ahn^3^, Keun-bae Song^1^, Youn-Hee Choi^1^

^1^Department of Preventive Dentistry, School of Dentistry, Kyungpook National University, Daegu, Republic of Korea

^2^Department of Dental Hygiene, Gumi University, Gumi, Republic of Korea

^3^Department of Health Convergence, Ewha Woman's University, Seoul, Republic of Korea

†These authors contributed equally to this work as co-first authors

**Corresponding Author**

Youn-Hee Choi, Department of Preventive Dentistry, School of Dentistry, Kyungpook National University, 2177 Dalgubeol-daero, Jung-gu, Daegu 41940, Korea

E-mail : [cyh1001@knu.ac.kr](mailto:cyh1001@knu.ac.kr)

**초록**

**목적:** 2007-2018년 한국성인의 인구사회학적 변수에 따른 무치악 유병률을 추정하고 변화추이를 분석하고자 하였다.

**방법:** 본 연구에서는 2007-2018년 국민건강영양조사(Korea National Health and Nutrition Examination Survey; KNHANES) 원시자료를 이용하여 분석하였으며, 상, 하악에 잔존치아가 전혀 없는 것과 잔존치근에 대해서 무치악으로 정의하였다. 무치악 유병률은 인구사회학적 변수에 따라 복합표본 빈도분석과 회귀분석을 수행하였고, 연령표준화된 무치악 유병률을 계산하기 위해 2005년 한국의 추계인구를 표준인구로 사용하였다.

**결과:** 2016-2018년 한국 노인의 무치악 유병률은 9.72%였으며, 연령표준화된 무치악 유병률의 변화는 2007 년 12.8 %에서 꾸준히 감소했다. 특히, 80대 인구의 경우 2007년 대비 2016년부터 2018년까지 약 20% 감소하였다. 성별에 따른 유병률은 여성에서 유의하게 감소하였으며, 교육수준에 따른 유병률은 최하위군에서 가장 큰 감소폭이 나타났지만 유병률 자체는 상위군에 비해 높았다.

**결론**: 한국 노인의 무치악 유병률은 시간이 지남에 따라 감소하였다. 그러나 여전히 무치악으로 어려움을 겪을 수 있는 교육수준이 낮은 노인과 남성에 대해선 지속적인 관심이 필요하다.

**키워드:** 구강건강, 국민건강영양조사, 무치악, 유병률, 치아상실

**서론**

전 세계적으로 치아 상실은 2017년 기준 2억6천7백만 개인의 구강 부담에 영향을 미칠 것으로 추정되며[1], 구강건강과 전신건강뿐만 아니라 삶의 질에도 상당한 악영향을 끼친다[2]. 치아의 기본기능인 저작, 발음, 심미 기능을 수행할 수 없도록 하여 상실감을 느끼게 하고[3], 더 나아가 영양상실, 체중저하를 유발하여 전신질환을 일으키며 심할 경우 사망에 이르게 한다[4-7]. 그러므로 치아를 상실하지 않고 건강하게 보존하는 것이 전신건강을 유지하는데 있어서 매우 중요하다. 치아상실은 대체로 40대부터 발생하기 시작하여 65세 이후 급격하게 증가하는 추세이며[8], 치아우식증이나 치주병, 악골 내 종양, 외상 등 다양한 원인으로 인해 발생한다[9,10]. 치아가 상실되면 치아의 역할을 할 수 있는 보철치료를 통하여 기능을 회복시킬 수 있다.

무치악이란 고정성 보철치료를 받지 않고 자연치아가 모두 상실된 상태를 뜻하는데, 국가나 지역에 따라 차이가 있지만 대체적으로 전 세계의 50세 이상 성인에서 높게 나타나고 젊은 연령층에서도 종종 관찰되어 집중해야 할 공중구강보건의 문제 중 하나로 여겨진다[11-13]. 지난 30년 동안 미국 등 선진국에서 무치악 유병률은 감소하였지만, 여전히 저소득층과 노인의 상당수는 무치악으로 인한 저작의 어려움을 겪고 있다[13,14].

인구가 고령화됨에 따라 세계적으로 노인인구와 구강건강의 연관성에 대해 관심이 높아졌다. 현재 각 나라별 무치악 유병률 추이 연구가 활발히 진행되고 있으며 나아가 다국 간의 비교도 이루어지고 있다. 대표적인 연구로 브라질의 국가구강건강조사 자료를 이용하여 무치악 유병률 및 동향을 살펴본 Cardoso, et al.[15]와 미국의 국가조사 자료를 이용한 Eklund & Burt[16]의 연구, 5개(호주, 캐나다, 칠레 뉴질랜드 및 미국) 국가 간 무치악 유병률 및 치아상실에 대한 교육 기반 사회불평등을 비교한 Elani, et al.[17]의 연구가 있다. 2016 년 Jeon & Ryu [3]는 제 6 차 국민 건강 영양 조사의 원 자료를 이용하여 치아 손실에 따른 노인의 구강 건강 행태를 보고했다. 그러나 2019 년 기준 65 세 이상 노인이 전체 인구의 14.9 %를 차지하고 있음에도 불구하고 우리나라에서 치아의 유병률과 치아 손실률에 대한 역학 연구가 부족하다. 또한 무치악 유병률의 중요한 예측인자인 인구사회학적 변수에 따라 추이를 분석한 미국의 Eklund & Burt[16]는 연구에서 전반적인 무치악 유병률의 감소에도 불구하고 저학력층과 저소득층에서 치아상실이 훨씬 더 높다고 보고하였다. 국내에서는 2014년부터 임플란트가 급여화 되었음에도 불구하고 지역/직장가입자보다 의료급여 수급권자인 저소득층의 이용률이 낮다는 Ryu & Jeon[18]의 연구결과가 있지만 급여화 된 시기 전후의 인구사회학적 변수에 따른 무치악 유병률 추이 연구는 아직 발표되지 않았다.

무치악 유병률 연구는 인구의 구강 건강 상태를 파악하고 진단할 수 있으며, 이를 개선하기 위한 정책을 수립할 수 있다. 따라서 본 연구에서는 2007-2018년(제4기-7기) 국민건강영양조사 원시 자료를 이용하여 연도별 인구사회학적 변수에 따른 한국의 무치악 유병률을 추정하고, 변화추이에 차이가 있는지 파악하고자 한다.

연구대상 및 방법

**1. 연구대상**

본 연구는 한국의 대표적인 건강 및 영양조사인 국민건강영양조사(Korea National Health and Nutrition Examination Survey; KNHANES) 구강검사가 실시된 제4기(2007-2009)부터 제5기(2010-2012), 제6기(2013-2015), 제7기(2016-2018)의 원시 자료를 이용하여 분석하였다.

연구대상자는 구강검사에 참여한 19세 이상의 성인을 대상으로 총 63,791명(2007년 2,925명, 2008년 6,681명, 2009년 7,403명, 2010년 6,206명, 2011년 6,039명, 2012년 5,757명, 2013년 5,439명, 2014년 5,118명, 2015년 5,024명, 2016-2018년 13,199명)의 자료를 사용하였다. 질병관리청에서 제4기부터 제6기까지는 연도별 원시자료를 제공하였으나 제7기 자료는 3년(2016-2018년) 자료가 통합되어 있는데, 이는 구강검사를 수행하는 공중보건치과의의 인력부족으로 전수조사를 하지 못했기 때문이다[19].

**2. 연구방법**

2.1 인구사회학적 변수

인구사회학적 변수로는 연령, 성별, 소득수준, 교육수준을 사용하였다. 연령은 19세 이상과 65세 이상, 그리고 30대, 40대, 50대, 60대, 70대, 80대 이상으로 구분하였으며, 2005년 발표된 추계 인구 비율을 적용한 연령표준화 근거에 따라 시행하였다. 성별은 남성과 여성으로 구분하였고, 소득수준은 “하”, “중하”, “중상”, “상”, 교육수준은 “초졸 이하”, “중졸”, “고졸”, “대졸 이상”으로 범주화하였다.

2.2 구강검사

국민건강영양조사의 구강검사는 설문조사와 검진조사로 나누어져 있으며, 교육훈련을 받은 공중보건치과의사가 이동검진센터에서 시행하였다. 검진조사 항목은 WHO 구강검사 지침을 기반으로 치아 및 보철물 상태, 치료 필요를 측정하였으며 치면상태부호 및 치료필요부호는 0부터 9까지 나누어 분류하였다[19].

2.3 무치악 정의

무치악의 조작적 정의는 구강 내 치아가 하나도 없는 상태이다[20]. 이 연구에서는 상, 하악 잔존치아가 없는 무치악 뿐만 아니라 잔존치근에 대해서도 상실치아로 정의하여 무치악 유병률의 추이를 비교, 분석하였다.

**3. 통계분석**

국민건강영양조사 자료는 복합표본설계 자료이므로 연도별 질병관리청에서 제시하는 가중치를 적용하여 통계분석을 실시하였다. 표준인구는 2005년 추계인구를 기준으로 연령구간은 19-29세, 30-39세, 40-49세, 50-59세, 60-69세, 70세 이상으로 구분하여 연령표준화를 수행하였다. 또한 해당 연도의 실제 유병률과 비교할 수 있게 연령표준화를 하지 않은 값과 병기하여 표기하였다.

무치악 환자의 연도별 일반적 특성 및 분포를 구하기 위해 복합표본 빈도분석을 수행하였다. 또한, 연도별 무치악 유병률 추이를 확인하기 위하여 연도별 인구사회학적 변수에 따른 무치악 유병률에 대해 회귀분석을 수행하였다.

통계 검정에서의 유의수준은 0.05로 정하였으며 통계 분석은 SAS 9.4 (SAS Institute, Cary, NC, USA) 통계프로그램을 이용하였다.

결과

**1. 연령에 따른 무치악 유병률 추이**

연령에 따른 무치악 유병률 추이를 분석한 결과, 연령표준화를 시행한 19세 이상 전체 대상자는 2007년 이후 전반적으로 감소하는 경향을 보이다가, 2012년과 2013년에 소폭 증가 후 다시 감소하는 추세가 나타났다(p<0.05)(Figure1). 노인 인구의 추이만 분석한 결과, 65세 이상 대상자의 무치악 유병률은 2007년부터 소폭 증가와 감소를 반복하지만 전반적으로 감소하는 추이였다(p<0.05)(Figure2). 80대 이상의 무치악 유병률에서는 2007년 유병률이 40%에 달했지만 2017년 대폭 감소하는 추이였다(p<0.05)(Figure2). 연령별 무치악 유병률 추이는 폭의 차이가 있지만, 2007년 대비 연령과 관계없이 시간의 흐름에 따라 감소하는 추세였으며, 또한 연령이 높을수록 무치악 유병률이 높은 것을 확인하였다. 모든 집단의 무치악 유병률이 2013년 소폭 상승 후 다시 감소하는 추세였고, 80대는 2013년을 기준으로 상승과 감소의 폭이 큰 것으로 나타났다(p<0.05)(Figure 1 & Figure 2).

**2. 성별에 따른 무치악 유병률 추이**

Figure3과 Figure4는 성별에 따른 무치악 유병률 추이를 나타낸 것이다. 연령표준화를 하지 않은 남성집단에서 유병률의 유의한 추이가 나타났지만, 연령표준화를 한 경우 소폭 증가와 감소를 반복하며 전반적으로 감소하는 경향이 있었다(Figure3). 여성집단의 경우 남성과 마찬가지로 증가와 감소를 반복하지만 남성에 비해 무치악 유병률이 눈에 띄게 감소하는 추이를 확인할 수 있었다. 여성집단은 연령표준화의 시행 유무와 관계없이 비슷한 추이를 나타냈으며 2007년-2017년 사이 소폭 증가와 감소를 반복하는 것으로 나타난다(p<0.05)(Figure4).

**3. 무치악 유병률 추이 분석 결과**

Table 1은 연령과 성별에 따른 무치악 유병률을 분석한 결과이다. 연령표준화를 시행한 19세 이상 대상자의 무치악 유병률은 2007년 1.88%로 이후 전반적으로 감소하는 경향을 보이며 2017년 1.31%로 소폭 감소하였다(p<0.05). 65세 이상 대상자의 무치악 유병률 추이 또한 증가와 감소를 반복하지만 2007년 12.80%에 비해 2016-2018년 9.72%로 25%감소하였다(p<0.05). 연령별 무치악 유병률 추이는 연령이 높을수록 유병률 또한 높았지만, 모든 연령에서 2007년 대비 2017년에 감소하였고 특히, 80대의 무치악 유병률이 37.30%에서 19.01%로 50%정도 대폭 감소하였다(p<0.05). 그리고 60대를 제외한 모든 집단의 무치악 유병률이 2013년 소폭 상승 후 다시 감소하는 추세였고, 80대는 2013년을 기준으로 상승과 감소의 폭이 큰 것으로 나타났다(p<0.05).

성별에 따른 무치악 유병률 추이는 남성과 여성 모두에서 2007년부터 2017년 사이 소폭 증가와 감소를 반복하는 것으로 나타난다. 2007년 연령표준화가 시행된 무치악 유병률은 남성이 1.75%, 여성이 1.94%로 여성이 높았으나 2017년 남성이 1.48%, 여성이 1.19%로 여성의 무치악 유병률이 더 낮은 것으로 나타났다. 또한 여성의 경우 유병률의 변화가 유의하였다(p<0.05)(Table 1).

연령표준화 시행 후 소득수준에 따른 무치악 유병률 분석 결과, 2007년 집단 간의 차이가 크지 않았지만 가장 상위집단에서는 2007년 대비 2017년 50%가 감소하였다. 그리고 2007년에서 2017년 사이 무치악 유병률 추이의 상승과 하락 폭은 하위 두 집단이 상위 두 집단에 비해 더 컸다(Table 2).

연령표준화를 시행 후 교육수준에 따른 무치악 유병률은 가장 낮은 집단이 2007년 2.97%에서 2017년 1.34%로 50% 이상 감소하였으나, 다른 집단에 비해 무치악 유병률이 높은 것으로 나타났다(p<0.05). 2007년 교육수준의 최하위 집단과 최상위 집단의 무치악 유병률 차이는 2.37%이고, 2017년 집단 간 차이는 0.76%로 시간의 흐름에 따라 무치악 유병률의 격차가 줄어든 것을 확인하였다(Table 2).

**토론**

치아상실은 구강건강의 지표로써 현재 국외에서는 무치악 유병률 추이 파악뿐만 아니라 다국 간의 비교가 이루어지고 있다[8,17]. 하지만 아시아의 주요 국가 중 하나인 한국의 무치악 유병률 추이에 대한 연구는 찾아보기 힘들다. 이에 본 연구에서는 2007-2018년(제4기-제7기) 국민건강영양조사 원시 자료를 이용하여 만 19세 이상 성인 인구의 무치악 유병률 추이를 파악하고자 하였다.

본 연구의 결과 인구사회학적 변수에 따른 무치악 유병률 추이는 증가와 감소를 반복하지만 2007년에 비해 2016-2018년 감소하였다. 또한 연령이 높을수록 무치악 유병률이 높았고 유병률 감소의 폭도 컸다. 연령별 추이 결과, 80대 에서는 2012년에서 2013년 9.57% 상승하였고, 2013년에서 2014년 13.89% 감소하였다. 이는 75세 이상 국민을 대상으로 2014년 07월 임플란트의 급여화가 시행된 시기와 일치한다. Ryu & Jeon[18]의 연구에 따르면 급여 임플란트 이용률이 2014년 1.2%, 2015년 3.2%, 2016년 4.9%로 증가하였다고 보고하였다. 가철성 국소의치의 지대치가 될 수 있는 치아가 충분하지 않을 때, 잔존 치아를 발치하여 총의치를 하는 대신 지대치 역할의 임플란트를 식립함으로써 가철성 국소의치를 사용할 수 있다. 이러한 경우에서 임플란트 급여화가 무치악 유병률 감소에 영향을 주었을 것으로 예상된다. 성별에 따른 무치악 유병률은 2007년 대비 2016-2018년 여성의 무치악 유병률 감소폭이 크지만, 급여 임플란트가 시행된 2014년 기준 남성은 감소하였고, 여성은 증가한 것으로 나타났다. 이 또한 여성보다 남성의 급여 임플란트 높은 이용률과 관련이 있을 것이다[18]. 소득수준에 따른 무치악 유병률의 경우 상위집단에서 무치악 유병률의 감소폭이 컸고, 하위집단일수록 무치악 유병률이 높았다. 이는 노인의 경우 무치악에 대해 사회경제적 요인에 큰 영향을 받으며, 전반적인 무치악 유병률의 감소에도 불구하고 저소득층의 치아상실 가능성이 높다고 보고한 Eklund SA, Burt[16]의 연구와도 일치한다. 또한 지역/직장가입자 보다 의료급여 수급권자들의 급여 임플란트 이용률이 낮다고 보고한 Ryu & Jeon[18]의 연구와 일치하는 결과이다. 임플란트가 급여화 되어도 그 외 치과치료의 비급여 항목이 많기 때문에 저소득층의 높은 무치악 유병률에 영향을 끼친 것으로 추정된다. 교육수준에 따른 무치악 유병률 추이는 Elani, et al.[17]의 미국, 호주, 캐나다, 뉴질랜드, 칠레 총 5개국을 비교한 연구와 마찬가지로 본 연구에서도 교육수준의 하위집단에 비해 상위집단의 무치악 유병률이 낮은 것으로 나타났다.

추가분석으로 기본기능을 수행할 수 없는 발거가 필요한 치아까지 무치악으로 포함하여 19세 이상 대상자를 분석해본 결과, 추이와 무치악 유병률은 비슷하였다. 80세 이상 고령층에서는 2016-2018년 기준, 구강 내 치아가 없는 무치악과 비교하였을 때 유병률이 0.43%의 차이를 보였는데, 이는 연령이 높을수록 기능을 상실한 치아를 치료하지 않거나 발거하지 않은 채 유지하고 있는 것으로 파악된다(data not shown). 이는 치아나 잇몸 등 구강 내의 문제로 인해 저작과 발음불편을 느끼는 구강기능제한율과 관련이 있을 것으로 예상되며, 실제로 2009-2019년 국민건강영양조사의 추이 결과 연령이 높을수록 구강기능제한율이 높게 나타났다[21].

고연령층일수록, 소득수준과 교육수준이 낮을수록 치아상실이 일어날 확률이 높고 그로 인한 무치악 유병률이 높다는 본 연구의 결과는 국외의 선행연구 결과와도 일치하는 것으로 확인되었다[8,22,23]. 인구사회학적 변수에 따른 무치악 유병률 추이 차이는 있지만 시간의 흐름에 따른 전반적 감소는 급여 임플란트 대상연령의 확대화와 본인 부담률 인하도 영향을 주었을 것으로 생각된다. 또한 국민건강영양조사 자료를 이용한 한국 장년층의 주관적 구강건강상태평가에 대한 연구 결과, 자신의 구강건강상태를 나쁘다고 보고한 장년층이 감소하는 추세로 구강건강에 대한 높은 관심 또한 긍정적 영향을 주었을 가능성이 있다[24]. 경제협력개발기구(Organisation for Economic Co-operation and Development;OECD) 국가 중 한국의 건강수준이 뒤지지 않지만, 치과의료에 대한 보장성이 상대적으로 취약하고 의료접근이 어려운 집단에서 의료제공 반비례 법칙에 의한 양극화는 심화되고 있어 개선할 필요가 있다[25]. 소득수준과 교육수준에 따른 무치악 유병률의 격차는 여전히 존재하고 있음을 고려해보면 저소득층과 저학력층에 속하는 국민을 위하여 구강건강 관련 접근성 및 급여화 확대와 같은 보편적인 정책이 지속적으로 추진되어야 할 것이다.

본 연구에는 몇 가지 제한점이 있다. 국민건강영양조사의 자료를 이용하였으나 구강조사는 구강검진과 설문으로 이루어진 단면연구로써 2013년 60대를 제외한 모든 집단의 무치악 유병률 증가에 대한 원인은 밝혀내지 못하였다. 따라서 이 결과에 대한 추후연구가 필요할 것으로 생각된다. 또한 치아상실과 관련있는 음주, 흡연 등과 같은 요인은 본 연구에서 다루지 않았다. 마지막으로 양로원, 요양 및 재활시설에서 생활하는 취약계층이 국민건강영양조사에서 제외되었다는 점이다.

그럼에도 불구하고 이 연구는 우리나라 국민을 대표하는 국민건강영양조사의 대규모 샘플을 이용하여 한국의 무치악 유병률 추이를 분석한 첫 연구라는 점이다. 또한 다국 간의 무치악 유병률 비교 연구에 아시아의 주요 국가인 한국의 참고자료로 활용이 가능하다는 것에 의의가 있다.

결론적으로, 2007년부터 2018년까지 만65세 이상의 무치악 유병률을 살펴본 바 12.80%에서 9.72%까지 시간의 흐름에 따라 전반적으로 감소하는 추세였으며, 인구사회학적 변수인 나이, 성별, 소득수준, 교육수준에 따른 집단별 유의한 변화추이를 확인할 수 있었다. 고연령층일수록, 소득수준과 교육수준이 낮을수록 무치악 유병률이 높지만, 2014년 시행된 임플란트 급여화가 무치악 유병률 감소에 관련이 있을 것으로 예상된다. 하지만 여전히 무치악으로 어려움을 겪는 집단이 있기에 후속적인 추적관찰 연구가 필요하다. 이에 본 연구의 결과가 치아상실 및 무치악 관련 구강건강에 대한 기초자료로 활용되기를 기대한다.

**윤리성명**

국민건강영양조사는 생명윤리법 제2조 제1호 및 동법 시행규칙 제2조 제2항 제1호에 따라 국가가 직접 공공복리를 위해 수행하는 연구에 해당하여 연구윤리심의위원회 심의를 받지 않고 수행 가능함.

**이해상충**

The authors have no conflicts of interest to declare for this study.

**감사의 말**

본 연구는 보건복지부의 재원으로 한국보건산업진흥원의 보건의료기술연구개발사업 지원에 의하여 이루어진 것임(과제고유번호: HI19C1218).

**참고문헌**

1.  [Bernabe](https://www.ncbi.nlm.nih.gov/pubmed/?term=Bernabe%20E%5BAuthor%5D&cauthor=true&cauthor_uid=32122215) E, [Marcenes](https://www.ncbi.nlm.nih.gov/pubmed/?term=Marcenes%20W%5BAuthor%5D&cauthor=true&cauthor_uid=32122215) W, [Hernandez](https://www.ncbi.nlm.nih.gov/pubmed/?term=Hernandez%20C%5BAuthor%5D&cauthor=true&cauthor_uid=32122215) C.R, [Bailey](https://www.ncbi.nlm.nih.gov/pubmed/?term=Bailey%20J%5BAuthor%5D&cauthor=true&cauthor_uid=32122215) J, [Abreu](https://www.ncbi.nlm.nih.gov/pubmed/?term=Abreu%20L%5BAuthor%5D&cauthor=true&cauthor_uid=32122215) L.G, [Alipour](https://www.ncbi.nlm.nih.gov/pubmed/?term=Alipour%20V%5BAuthor%5D&cauthor=true&cauthor_uid=32122215) V, et al. Global, Regional, and National Levels and Trends in Burden of Oral Conditions from 1990 to 2017: A Systematic Analysis for the Global Burden of Disease 2017 Study. J Dent Res 2020;99:362–373.

2. McGrath C, Bedi R. Measuring the impact of oral health on life quality in two national surveys - functionalist versus hermeneutic approaches. Community Dent Oral Epidemiol 2002;30:254-259.

3. Jeon MJ, Ryu SY. Oral health and behavior by tooth loss: The Sixth Korea National Health and Nutrition Examination Survey. Journal of KOEN 2016;10:259-268.

4. Wu LL, Cheung KY, Lam PYP, Gao XL. Oral health indicators for risk of malnutrition in elders. J Nutr Health Aging 2018;22:254-261

5. Song IS, Han KD, Ryu JJ, Park JB. Association between underweight and tooth loss among Korean adults. Sci Rep 2017;7:41524-41530.

6. Kim EK, Lee SK, Jung YS, Lee HK, Song KB, Choi YH. Associations between remaining teeth and salivary flow, activity of daily living, and cognitive impairment among the elderly in a rural area: A pilot study. J Korean Acad Oral Health 2016;40:43-48.

7. Hirotomi T, Yoshihara A, Ogawa H, Miyazaki H. Number of teeth and 5-year mortality in an elderly population. Community Dent Oral Epidemiol 2015;43:226-231.

8. Kim HN, Ha TG, Kim MJ, Jun EJ, Jeong SH, Kim JB. Factors related to number of present teeth in Korean elderly adults aged 55-84 years. Int J Dent Hyg 2016;14:151-158.

9. Cooper LF. The current and future treatment of edentulism. J Prosthodont 2009;18:116-122.

10. Lee SK, Lee KW, Chang KW. Reasons for extracted permanent teeth in Korean population. J Korean Acad Oral Health 2001;25:139-165.

11. Nordenram G, Davidson T, Gynther G, Helgesson G, Hultin M, Jemt T, et al. Qualitative studies of patients' perceptions of loss of teeth, the edentulous state and prosthetic rehabilitation: a systematic review with meta synthesis. Acta Odontol Scand 2013;71:937-951.

12. Peltzer K, Hewlett S, Yawson AE, Moynihan P, Preet R, Wu F. Prevalence of loss of all teeth (edentulism) and associated factors in older adults in China, Ghana, India, Mexico, Russia and South Africa. Int J Environ Res Public Health 2014;11:11308-11324.

13. Peres MA, Barbato PR, Sandra Reis SC, Freitas CH, Antunes JL. Tooth loss in Brazil: analysis of the 2010 Brazilian oral health survey. Rev Saude Publica 2014;47:78-89.

14. Fabio C, Guglielmo C, Laura S, Viviana CA, Maria GC. The burden of tooth loss in Italian elderly population living in nursing homes. BMC Geriatr 2018;18:76-84.

15. Cardoso M, Balducci I, Telles D, Lourenco EJ, Junior LN. Edentulism in Brazil: trends, projections and expectations until 2040. Cien Saude Colet 2016;21:1239-1245.

16. Eklund SA, Burt BA. Risk factors for total tooth loss in the United States; longitudinal analysis of national data. J Public Health Dent 1994;54:5-14.

17. Elani HW, Harper S, Thomson WM, Espinoza IL, Mejia GC, Ju X, et al. Social inequalities in tooth loss: A multinational comparison. Community Dent Oral Epidemiol 2017;45:266-274.

18. Ryu JI, Jeon JE. Utilization rate of dental implant for elderly in National Health Insurance in Korea. J Kor Dent Assoc 2019;57:496-503.

19. Woo GJ, Lee HR, Kim YJ, Kim HJ, Park DY, Kim JB. Data resource profile: oral examination of the Korea National Health and Nutrition Examination Survey. J Korean Acad Oral Health 2018;42:101-108.

20. McGarry TJ, Nimmo A, Skiba JF, Ahlstrom RH, Smith CR, Koumjian JH. Classification system for complete edentulism. J Prosthet Dent 1999;8;27-39.

21. Korea Disease Control and Prevention Agency. Korea Health Statistics 2019: Korea National Health and Nutrition Examination Survey (KNHANES Ⅷ-1). Chungbuk:Korea Disease Control and Prevention Agency;2020:165-167.

22. Emami E, Souza RF, Kabawat M, Feine JS. The impact of edentulism on oral and general health. Int J Dent 2013;2013:498305-498311.

23. Wu B, Hybels C, Liang J, Landerman L, Plassman B. Social stratification and tooth loss among middle‐aged and older Americans from 1988 to 2004. Community Dent Oral Epidemiol 2014;42:495-502.

24. Kim SY, Kim NH. Change over a period of 9 years in self-reported oral health of a middle-aged population using 4-6th KNHANES data. J Korean Acad Oral Health 2019;43:55-55.

25. Ministry of Health and Welfare. Health care in Korea from OECD statistics. Sejong:Ministry of Health and Welfare;2019:3-4.

| \| 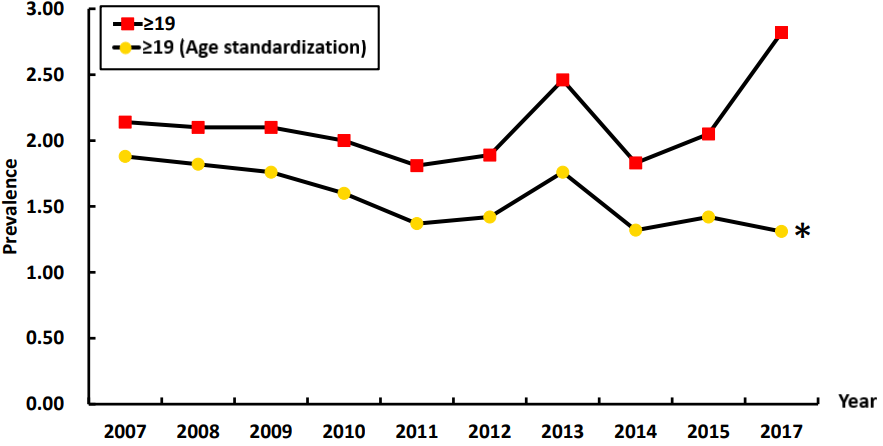 \| \| --- \| \| Figure 1. Trend analysis of edentulism more than the age of 19 in KNHANES *p-value for trend test < 0.05  Prevalence of 2017 was calculated from 2016-2018 data.  Crude prevalence means real magnitude of edentulism at the time of the survey. \| |
| --- | --- | --- |
| 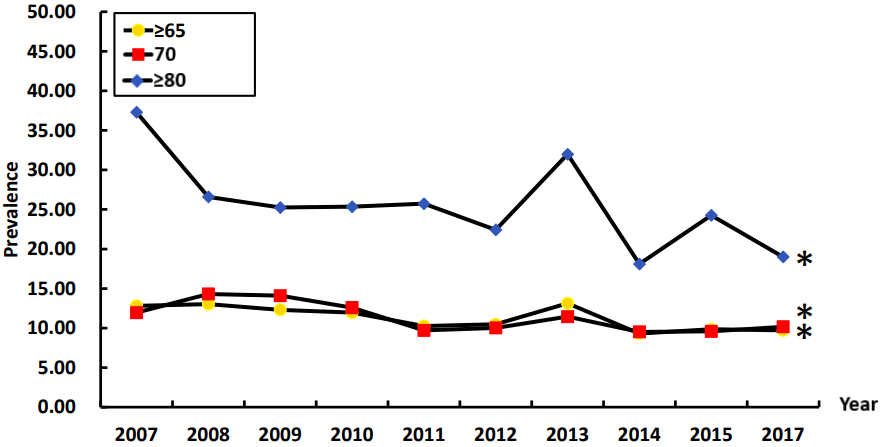 |
| Figure 2. Trend analysis of edentulism more than the age of 65 in KNHANES  *p-value for trend test < 0.05  Prevalence of 2017 was calculated from 2016-2018 data. |

|  |
| --- |
| \|  \| \| --- \| \| \| 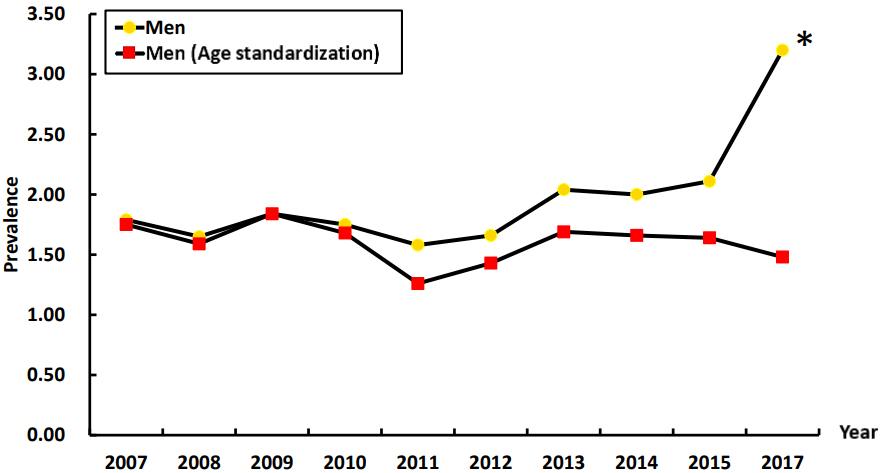 \| \| --- \| \| Fig ３. Trend analysis of edentulism of men in KNHANES  *p-value for trend test < 0.05  Prevalence of 2017 was calculated from 2016-2018 data.  Crude prevalence means real magnitude of edentulism at the time of the survey. \| \|  \| 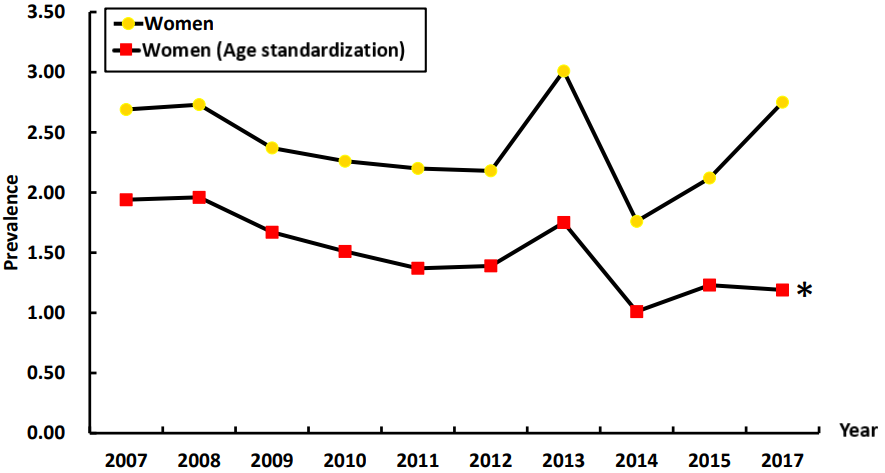 \| \| --- \| \| Figure 4. Trend analysis of edentulism of women in KNHANES  *p-value for trend test < 0.05  Prevalence of 2017 was calculated from 2016-2018 data.  Crude prevalence means real magnitude of edentulism at the time of the survey. \| |
|  |

Table 1. Changes in edentulism prevalence according to age and sex

|  | 2007 | | 2008 | | 2009 | | 2010 | | 2011 | | 2012 | | 2013 | | 2014 | | 2015 | | ^†^2017 | | Trend test |
| --- | --- | --- | --- | --- | --- | --- | --- | --- | --- | --- | --- | --- | --- | --- | --- | --- | --- | --- | --- | --- | --- |
|  | n  (%) | SE | n  (%) | SE | n  (%) | SE | n  (%) | SE | n  (%) | SE | n  (%) | SE | n  (%) | SE | n  (%) | SE | n  (%) | SE | n  (%) | SE | p-value |
| ≥19 | 2925  (2.14) | 0.35 | 6681  (2.10) | 0.19 | 7403  (2.10) | 0.19 | 6206  (2.00) | 0.21 | 6039  (1.81) | 0.21 | 5757  (1.89) | 0.21 | 5439  (2.46) | 0.24 | 5118  (1.83) | 0.20 | 5024  (2.05) | 0.20 | 13199  (2.82) | 0.23 | 0.2412 |
| ≥19  (standardization) | 2925  (1.88) | 0.26 | 6681  (1.82) | 0.14 | 7403  (1.76) | 0.14 | 6206  (1.60) | 0.15 | 6039  (1.37) | 0.14 | 5757  (1.42) | 0.13 | 5439  (1.76) | 0.15 | 5118  (1.32) | 0.14 | 5024  (1.42) | 0.13 | 13199  (1.31) | 0.09 | **0.0069** |
| ≥65 | 727  (12.80) | 1.92 | 1497  (13.04) | 1.11 | 1658  (12.31) | 1.05 | 1396  (11.95) | 1.27 | 1521  (10.26) | 1.05 | 1559  (10.48) | 1.13 | 1289  (13.13) | 1.15 | 1399  (9.30) | 0.93 | 1349  (9.85) | 0.99 | 3426  (9.72) | 0.72 | **0.0140** |
| Age  (yr) |  |  |  |  |  |  |  |  |  |  |  |  |  |  |  |  |  |  |  |  |  |
| 50 | 494  (1.27) | 0.58 | 1116  (1.06) | 0.33 | 1235  (1.03) | 0.36 | 1164  (1.03) | 0.26 | 1168  (1.22) | 0.37 | 1087  (0.65) | 0.31 | 1021  (1.24) | 0.42 | 967  (0.85) | 0.37 | 1029  (1.09) | 0.49 | 2493  (0.58) | 0.21 | 0.0940 |
| 60 | 479  (4.13) | 0.96 | 1063  (4.35) | 0.77 | 1196  (4.05) | 0.68 | 1011  (3.45) | 0.72 | 1033  (2.43) | 0.62 | 1028  (4.15) | 0.73 | 870  (3.63) | 0.72 | 890  (3.37) | 0.62 | 936  (2.75) | 0.66 | 2256  (3.08) | 0.44 | 0.0611 |
| 70 | 371  (11.95) | 1.64 | 754  (14.31) | 1.52 | 859  (14.10) | 1.60 | 734  (12.58) | 1.57 | 828  (9.71) | 1.18 | 879  (10.02) | 1.45 | 669  (11.45) | 1.54 | 733  (9.51) | 1.32 | 697  (9.59) | 1.41 | 1746  (10.16) | 0.93 | **0.0166** |
| ≥80 | 87  (37.30) | 7.89 | 195  (26.59) | 4.04 | 205  (25.25) | 3.48 | 159  (25.35) | 4.41 | 206  (25.73) | 3.92 | 198  (22.42) | 3.78 | 201  (31.99) | 3.84 | 220  (18.10) | 2.73 | 200  (24.27) | 3.22 | 621  (19.01) | 1.76 | **0.0416** |
| Gender |  |  |  |  |  |  |  |  |  |  |  |  |  |  |  |  |  |  |  |  |  |
| Men | 1215  (1.79) | 0.36 | 2787  (1.65) | 0.25 | 3207  (1.84) | 0.25 | 2678  (1.75) | 0.27 | 2569  (1.58) | 0.23 | 2389  (1.66) | 0.28 | 2336  (2.04) | 0.26 | 2133  (2.00) | 0.28 | 2187  (2.11) | 0.29 | 5793  (3.20) | 0.31 | **0.0138** |
| Men (standardization) | 1215  (1.75) | 0.34 | 2787  (1.59) | 0.22 | 3207  (1.84) | 0.22 | 2678  (1.68) | 0.23 | 2569  (1.26) | 0.19 | 2389  (1.43) | 0.22 | 2336  (1.69) | 0.21 | 2133  (1.66) | 0.22 | 2187  (1.64) | 0.21 | 5793  (1.48) | 0.14 | 0.3846 |
| Women | 1710  (2.69) | 0.52 | 3894  (2.73) | 0.28 | 4196  (2.37) | 0.27 | 3528  (2.26) | 0.28 | 3470  (2.20) | 0.32 | 3368  (2.18) | 0.26 | 3103  (3.01) | 0.38 | 2985  (1.76) | 0.24 | 2837  (2.12) | 0.32 | 7406  (2.75) | 0.30 | 0.6240 |
| Women (standardization) | 1710  (1.94) | 0.32 | 3894  (1.96) | 0.18 | 4196  (1.67) | 0.18 | 3528  (1.51) | 0.18 | 3470  (1.37) | 0.17 | 3368  (1.39) | 0.16 | 3103  (1.75) | 0.20 | 2985  (1.01) | 0.13 | 2837  (1.23) | 0.19 | 7406  (1.19) | 0.12 | **0.0046** |
| n :　Unweighed, (%) :　Weighted proportions.  ^†^2017: Analysed of data from 2016-2018  Crude prevalence means real magnitude of edentulism at the time of the survey. | | | | | | | | | | | | | | | | | | | | | |

Table 2. Changes in edentulism prevalence according to income level and education level

|  | 2007 | | 2008 | | 2009 | | 2010 | | 2011 | | 2012 | | 2013 | | 2014 | | 2015 | | ^†^2017 | | Trend test |
| --- | --- | --- | --- | --- | --- | --- | --- | --- | --- | --- | --- | --- | --- | --- | --- | --- | --- | --- | --- | --- | --- |
|  | n  (%) | SE | n  (%) | SE | n  (%) | SE | n  (%) | SE | n  (%) | SE | n  (%) | SE | n  (%) | SE | n  (%) | SE | n  (%) | SE | n  (%) | SE | p-value |
| Income levle |  |  |  |  |  |  |  |  |  |  |  |  |  |  |  |  |  |  |  |  |  |
| Poorest | 616  (6.21) | 0.93 | 1368  (7.45) | 0.77 | 1581  (6.48) | 0.69 | 1252  (6.37) | 0.90 | 1212  (7.14) | 0.83 | 1104  (5.96) | 0.82 | 1120  (8.26) | 0.86 | 1034  (6.15) | 0.80 | 996  (6.70) | 0.86 | 2641  (9.19) | 0.84 | 0.1614 |
| Poor | 727  (1.84) | 0.47 | 1689  (1.50) | 0.28 | 1707  (2.49) | 0.45 | 1551  (1.54) | 0.35 | 1553  (1.27) | 0.32 | 1469  (2.18) | 0.39 | 1419  (1.38) | 0.32 | 1277  (2.26) | 0.47 | 1207  (2.73) | 0.54 | 3209  (2.70) | 0.34 | 0.1124 |
| Rich | 704  (0.63) | 0.24 | 1692  (0.97) | 0.26 | 2007  (0.78) | 0.19 | 1680  (0.89) | 0.27 | 1635  (0.58) | 0.15 | 1483  (0.39) | 0.13 | 1368  (1.48) | 0.32 | 1429  (0.75) | 0.20 | 1351  (0.77) | 0.24 | 3602  (1.13) | 0.20 | 0.4168 |
| Richest | 726  (0.90) | 0.34 | 1722  (0.94) | 0.23 | 2020  (0.50) | 0.16 | 1639  (0.68) | 0.18 | 1579  (0.69) | 0.22 | 1615  (0.79) | 0.27 | 1492  (1.11) | 0.32 | 1351  (0.47) | 0.21 | 1436  (0.42) | 0.17 | 3709  (0.57) | 0.13 | 0.2126 |
| Income levle  (standardization) |  |  |  |  |  |  |  |  |  |  |  |  |  |  |  |  |  |  |  |  |  |
| Poorest | 616  (1.75) | 0.25 | 1368  (2.39) | 0.28 | 1581  (1.84) | 0.20 | 1252  (1.90) | 0.26 | 1212  (1.91) | 0.31 | 1104  (1.35) | 0.19 | 1120  (2.27) | 0.33 | 1034  (1.30) | 0.17 | 996  (1.92) | 0.36 | 2641  (1.72) | 0.17 | 0.3986 |
| Poor | 727  (1.95) | 0.48 | 1689  (1.36) | 0.25 | 1707  (2.07) | 0.34 | 1551  (1.56) | 0.34 | 1553  (1.18) | 0.25 | 1469  (1.65) | 0.28 | 1419  (1.01) | 0.24 | 1277  (1.57) | 0.31 | 1207  (1.82) | 0.38 | 3209  (1.24) | 0.17 | 0.2869 |
| Rich | 704  (1.07) | 0.42 | 1692  (1.62) | 0.40 | 2007  (1.29) | 0.31 | 1680  (1.34) | 0.40 | 1635  (0.78) | 0.23 | 1483  (0.58) | 0.18 | 1368  (1.96) | 0.39 | 1429  (0.98) | 0.25 | 1351  (0.87) | 0.25 | 3602  (0.93) | 0.16 | 0.4255 |
| Richest | 726  (1.39) | 0.60 | 1722  (1.87) | 0.39 | 2020  (0.99) | 0.26 | 1639  (1.08) | 0.29 | 1579  (1.16) | 0.35 | 1615  (1.18) | 0.37 | 1492  (1.85) | 0.51 | 1351  (0.66) | 0.25 | 1436  (0.62) | 0.25 | 3709  (0.77) | 0.18 | 0.0887 |
| Education level |  |  |  |  |  |  |  |  |  |  |  |  |  |  |  |  |  |  |  |  |  |
| None or elementary school | 921  (8.02) | 1.30 | 1981  (8.87) | 0.75 | 2052  (7.80) | 0.68 | 1568  (6.79) | 0.81 | 1545  (7.50) | 0.88 | 1424  (6.01) | 0.77 | 1226  (8.62) | 1.00 | 1120  (5.98) | 0.78 | 1095  (7.46) | 0.82 | 2629  (8.98) | 0.76 | 0.8755 |
| Middle School | 315  (1.83) | 0.69 | 749  (1.91) | 0.51 | 820  (1.84) | 0.52 | 662  (2.18) | 0.54 | 655  (1.46) | 0.46 | 590  (1.96) | 0.53 | 526  (2.20) | 0.69 | 496  (2.51) | 0.68 | 510  (3.77) | 1.17 | 1233  (3.58) | 0.63 | **0.0057** |
| High school | 942  (0.53) | 0.22 | 2253  (0.13) | 0.06 | 2560  (0.42) | 0.11 | 2028  (0.46) | 0.16 | 1954  (0.28) | 0.10 | 1815  (0.69) | 0.26 | 1780  (0.88) | 0.24 | 1518  (0.74) | 0.18 | 1522  (0.59) | 0.19 | 4028  (1.20) | 0.20 | **0.0082** |
| College or University | 703  (0.10) | 0.08 | 1674  (0.35) | 0.17 | 1911  (0.19) | 0.12 | 1868  (0.25) | 0.09 | 1746  (0.32) | 0.14 | 1633  (0.06) | 0.03 | 1571  (0.31) | 0.13 | 1467  (0.39) | 0.20 | 1498  (0.35) | 0.15 | 4676  (0.36) | 0.10 | 0.1243 |
| Education level  (standardization) |  |  |  |  |  |  |  |  |  |  |  |  |  |  |  |  |  |  |  |  |  |
| None or elementary school | 921  (2.97) | 0.48 | 1981  (2.25) | 0.20 | 2052  (1.98) | 0.20 | 1568  (1.57) | 0.19 | 1545  (1.79) | 0.26 | 1424  (2.56) | 0.31 | 1226  (1.93) | 0.28 | 1120  (1.19) | 0.16 | 1095  (1.55) | 0.23 | 2629  (1.34) | 0.12 | **0.0206** |
| Middle School | 315  (1.62) | 0.56 | 749  (1.13) | 0.34 | 820  (1.38) | 0.37 | 662  (1.34) | 0.35 | 655  (0.82) | 0.27 | 590  (0.97) | 0.25 | 526  (1.03) | 0.32 | 496  (1.19) | 0.28 | 510  (2.14) | 0.99 | 1233  (1.13) | 0.22 | 0.9360 |
| High school | 942  (1.73) | 0.61 | 2253  (0.42) | 0.22 | 2560  (1.10) | 0.32 | 2028  (1.22) | 0.42 | 1954  (0.65) | 0.26 | 1815  (1.19) | 0.42 | 1780  (1.26) | 0.33 | 1518  (1.33) | 0.32 | 1522  (0.80) | 0.24 | 4028  (1.04) | 0.18 | 0.8361 |
| College or University | 703  (0.60) | 0.43 | 1674  (0.62) | 0.26 | 1911  (0.49) | 0.23 | 1868  (0.92) | 0.39 | 1746  (0.97) | 0.48 | 1633  (0.18) | 0.09 | 1571  (0.83) | 0.36 | 1467  (0.64) | 0.27 | 1498  (1.08) | 0.44 | 4676  (0.58) | 0.15 | 0.6502 |
| n :　Unweighed, (%) :　Weighted proportions.  ^†^2017: Analysed of data from 2016-2018  Crude prevalence means real magnitude of edentulism at the time of the survey. | | | | | | | | | | | | | | | | | | | | | |
